# Health Care Costs Associated With Muscle Weakness: A UK Population-Based Estimate

**DOI:** 10.1007/s00223-018-0478-1

**Published:** 2018-09-22

**Authors:** Rafael Pinedo-Villanueva, Leo D. Westbury, Holly E. Syddall, Maria T. Sanchez-Santos, Elaine M. Dennison, Sian M. Robinson, Cyrus Cooper

**Affiliations:** 1 Musculoskeletal Epidemiology, Botnar Research Centre, Nuffield Department of Orthopaedics, Rheumatology and Musculoskeletal Sciences, University of Oxford, Oxford, UK; 2 MRC Lifecourse Epidemiology Unit, University of Southampton, Southampton, UK; 3 Victoria University of Wellington, Wellington, New Zealand; 4 NIHR Southampton Biomedical Research Centre, University of Southampton and University Hospital Southampton NHS Foundation Trust, Southampton, UK; 5 NIHR Musculoskeletal Biomedical Research Centre, University of Oxford, Oxford, UK

**Keywords:** Sarcopenia, Muscle weakness, Health care costs, Ageing

## Abstract

Sarcopenia and muscle weakness are responsible for considerable health care expenditure but little is known about these costs in the UK. To address this, we estimated the excess economic burden for individuals with muscle weakness regarding the provision of health and social care among 442 men and women (aged 71–80 years) who participated in the Hertfordshire Cohort Study (UK). Muscle weakness, characterised by low grip strength, was defined according to the Foundation for the National Institutes of Health criteria (men < 26 kg, women < 16 kg). Costs associated with primary care consultations and visits, outpatient and inpatient secondary care, medications, and formal (paid) as well as informal care for each participant were calculated. Mean total costs per person and their corresponding components were compared between groups with and without muscle weakness. Prevalence of muscle weakness in the sample was 11%. Mean total annual costs for participants with muscle weakness were £4592 (CI £2962–£6221), with informal care, inpatient secondary care and primary care accounting for the majority of total costs (38%, 23% and 19%, respectively). For participants without muscle weakness, total annual costs were £1885 (CI £1542–£2228) and their three highest cost categories were informal care (26%), primary care (23%) and formal care (20%). Total excess costs associated with muscle weakness were £2707 per person per year, with informal care costs accounting for 46% of this difference. This results in an estimated annual excess cost in the UK of £2.5 billion.

**Electronic supplementary material:**

The online version of this article (10.1007/s00223-018-0478-1) contains supplementary material, which is available to authorized users.

## Introduction

Sarcopenia is characterised by the aggressive loss of skeletal muscle mass and strength with age [1]. It is associated with increased risk of functional impairment, poor health-related quality of life, physical frailty and premature death [2]. Sarcopenia is now regarded as a specific disease according to the International Classification of Diseases [3].

There is currently no consensus algorithm for defining sarcopenia. Low grip strength in older age is a risk factor for disability and mortality and a key component of sarcopenia [4]. For example, the European Working Group on Sarcopenia in Older People (EWGSOP) defines sarcopenia as having weak grip strength or slow gait speed in combination with low lean mass [5] and the Foundation for the National Institutes of Health (FNIH) Sarcopenia Project has defined sarcopenia as having weak grip strength and a low appendicular lean mass, adjusted for BMI [6].

Sarcopenia and muscle weakness are responsible for considerable health care expenditure. Annual direct medical costs attributable to sarcopenia were estimated at around $18.5 billion in the United States in 2000, representing 1.5% of total direct health care costs [7]. A cost-of-illness study in the Czech Republic, comprising 689 participants, aged 70 years and over, suggested that muscle weakness, indicated by low grip strength, was associated with increased yearly health care costs of €564 per person [8].

To our knowledge, no previous studies have estimated the economic costs of sarcopenia or muscle weakness in the UK. To address this gap, we have estimated the excess economic burden (regarding the provision of health and social care) for individuals with muscle weakness (identified by low grip strength according to the FNIH thresholds: men < 26 kg, women < 16 kg) using data from community-dwelling men and women (aged 71–80 years) who participated in the Hertfordshire Cohort Study (HCS).

## Methods

### The Hertfordshire Cohort Study

The Hertfordshire Cohort Study comprises 1579 men and 1418 women born in Hertfordshire in 1931–1939 and who still lived there in 1998–2004 when they attended a clinic visit and a nurse-administered home interview for a detailed characterisation of their sociodemographic, lifestyle and clinical characteristics. This study has been described in detail previously [9]. Smoking status and level of physical activity (Dallosso questionnaire [10]) were ascertained by a nurse-administered questionnaire. Social class was coded from the 1990 OPCS Standard Occupational Classification (SOC90) unit group for occupation [11].

Of the 2997 baseline participants, 966 participants from East Hertfordshire had a dual-energy X-ray absorptiometry (DXA) scan at baseline. In 2004, 642 of them were recruited to a clinical follow-up study. In 2011, 591 were invited to participate in a further follow-up study; 443 agreed to participate [12]. Smoking status (ever/never) and whether participants were suffering from a limiting long-term illness were ascertained through a nurse-administered questionnaire. Questions to ascertain Strawbridge frailty [13] and the Townsend disability scale [14], a score to reflect the difficulty in performing activities of daily living, were also asked. Participants were asked whether a doctor had told them that they had any of the following conditions: high blood pressure, diabetes, lung disease (such as asthma, chronic bronchitis, emphysema or COPD), rheumatoid arthritis, multiple sclerosis, thyroid disease, vitiligo, depression, Parkinson’s disease, heart disease (such as a heart attack, angina or heart failure), peripheral arterial disease (such as claudication), stroke, osteoporosis or cancer. The number of comorbidities was used as a marker of comorbidity.

### Ascertainment of Anthropometry and Grip Strength at Clinic

Height was measured to the nearest 0.1 cm using a Harpenden pocket stadiometer (Chasmors Ltd., London, UK) and weight to the nearest 0.1 kg on a SECA floor scale (Chasmors Ltd., London, UK). Body mass index (BMI) was calculated as weight divided by height^2^ (kg/m^2^). Grip strength was assessed three times for each hand using a Jamar dynamometer; the highest measurement was used for analysis. Muscle weakness, characterised by low grip strength, was defined according to FNIH criteria (men < 26 kg, women < 16 kg). This approach accords with that previously implemented in an analysis of data from the Survey of Health, Ageing and Retirement in Europe (SHARE) [8].

### Ascertainment of Health and Social Care Use

The number of primary care visits in the previous month to and from general practitioners (GPs), nurses and physiotherapists was ascertained from the nurse-administered questionnaire. The number of outpatient secondary care visits in the previous year to and from rheumatologists, orthopaedic surgeons, accident and emergency doctors, physiotherapists and podiatrists was also obtained. Medical procedures performed during the previous year were reported by participants. Participants provided details of all prescriptions as open text, detailing quantity, frequency and duration. Participants were asked whether they had received formal (paid) care or informal care at home in the past year, including questions about the type, frequency and provider. Please see Online Appendix 1 for further details.

### Derivation of Health and Social Care Costs

Costs were calculated by multiplying quantities of resource use by their respective unit costs. For GP and nurse consultations, the official publication of Unit Costs of Health and Social Care 2015 [15] was used. This was also the source of unit costs for outpatient secondary care consultations with physiotherapists and podiatrists, whilst for rheumatologists, orthopaedic surgeons and A&E doctors/traumatologists, unit costs were obtained from the 2014–2015 national reference costs for outpatient attendances [16].

For hospitalisations, operation-specific weighted averages of NHS reference costs were used as unit costs. These were calculated by identifying the set of health care resource groups (HRGs), which group health care activities demanding similar levels of resources, that relate to operations reported by participants. Weighted average unit costs were calculated using the activity reported for those HRGs on patients 70 years of age or older in national admitted patient care statistics [17] combined with hospital costs reported in the NHS National Schedule of Reference Costs tariff for 2014–2015 [18].

Unit costs for prescriptions were obtained from the national report on the net ingredient cost of all prescriptions dispensed in England [19]. The net ingredient cost per quantity (such as individual tablet or capsule) was used, matching the patient-reported prescription upon which the annual quantity of medication was estimated.

Unit costs for formal care were obtained from the Unit Costs of Health and Social Care 2015 publication [15], accounting for the differences between Social Services and those privately provided. For informal care, following the opportunity cost method [20], we used national average wages as an estimate for the value in monetary terms of the unpaid time dedicated to providing care at home. Where the care was provided by the participant’s children, we assumed they would have been employed and hence applied the average wage, whereas when provided by other friends of the family, we used the minimum wage. Online Appendix 2 details the unit costs used for primary and secondary care visits and consultations, hospitalisations, as well as formal and informal care. All costs estimated are in 2015 British pounds.

### Statistical Methods

Data were described using summary statistics. Differences in participant characteristics between individuals with and without muscle weakness were examined using *t* tests, *χ*^2^ tests, Fisher’s exact tests and Wilcoxon rank-sum tests as appropriate; normality was assessed by visual inspection of histograms.

Mean total costs per patient during 1 year and their corresponding cost components were compared between individuals with and without muscle weakness, the difference between them considered as the excess economic burden associated with muscle weakness. The estimated burden was then combined with the observed prevalence to produce an estimate for the economic burden of the disease in the UK.

Patient-level excess economic burden was modelled adjusting for social class and for variables ascertained in 2011 that differed significantly between the two groups to test their impact on the statistical significance of muscle weakness as a determinant of excess costs. A multivariate generalised linear (GL) model was estimated, with the family distribution identified using the Modified Park Test and the link function based on the Akaike and Bayesian information criteria.

Missing data for variables with a frequency equal to or lower than 1% was addressed by single imputation using regression and mean imputation. In the case of medication, missing quantity, frequency and/or duration was addressed by applying multiple imputation by chained equations methods [21] and 40 datasets were generated.

Sensitivity analyses were conducted using two alternative muscle strength criteria: < 30 kg and < 20 kg for men and women, respectively, proposed by Lauretani [22]; and the lowest decile of grip strength within each sex group. To produce an estimate of prevalence, we used the number of individuals classified as having muscle weakness over the total number of subjects reporting grip strength in the study. Statistical difference between excess costs by groups using these alternative criteria was assessed via the GL model described above.

The analysis sample consisted of the 442 participants (221 men and 221 women) with non-missing values for grip strength. Healthy participant effects were assessed by comparing HCS baseline participant characteristics between this analysis sample of 442 participants and the group of 2555 participants who attended the HCS baseline clinic but were not included in the analysis sample. All analyses were conducted in Stata 15 (StataCorp. 2017. Stata Statistical Software: Release 15. College Station, TX: StataCorp LLC).

## Results

### Participant Characteristics

The characteristics of the 442 participants according to muscle strength are presented in Table 1. Median (lower quartile, upper quartile) age of the sample at the 2011 follow-up was 75.5 (73.5, 77.9) years. Overall, 49 (11.1%) participants (20 [9.0%] men and 29 [13.1%] women) had muscle weakness. On average, participants with muscle weakness were older (*p* = 0.008) and had higher scores for Townsend disability (*p* < 0.001) compared to those without muscle weakness. Having previously smoked, a limiting long-term illness and Strawbridge frailty were each more common among individuals with muscle weakness compared to those without (*p* < 0.01 for all associations). There were no statistically significant associations between muscle strength and gender or BMI.

**Table 1:** Characteristics of the 442 Hertfordshire Cohort Study participants according to muscle strength at the 2011 follow-up

N (%)	Without muscle weakness (n = 393)	Muscle weakness (n = 49)	*p*
Characteristics at HCS baseline (1998–2004)			
Age (years)**	64.5 (62.5, 67.0)	65.9 (63.9, 68.0)	**0.004**
BMI (kg/m^2^)*	26.7 (4.1)	27.5 (3.6)	0.131
Ever smoked	181 (46.1%)	30 (61.2%)	**0.045**
Physical activity (Dallosso)*	63.7 (13.5)	61.5 (15.8)	0.301
Social class (manual)	208 (54.5%)	34 (69.4%)	**0.047**
Characteristics at follow-up (2011)			
Age (years)**	75.2 (73.3, 77.7)	76.6 (74.3, 78.7)	**0.008**
Gender (women)	192 (48.9%)	29 (59.2%)	0.173
BMI (kg/m^2^)*	28.1 (4.6)	28.5 (4.3)	0.508
Ever smoked	182 (46.3%)	33 (67.3%)	**0.005**
Limiting long-term illness	94 (23.9%)	21 (42.9%)	**0.004**
Strawbridge overall frailty	52 (13.3%)	14 (28.6%)	**0.005**
Townsend disability score**	2.0 (0.0, 4.0)	5.0 (2.0, 8.0)	< **0.001**
Number of comorbidities^+^			0.303
0	91 (23.2%)	6 (12.2%)	
1	122 (31.0%)	19 (38.8%)	
2	102 (26.0%)	11 (22.4%)	
3	40 (10.2%)	6 (12.2%)	
4 or more	38 (9.7%)	7 (14.3%)	

Values are given in bold at *p* < 0.05

*Mean (SD); *p* values derived using t tests

**Median (lower quartile, upper quartile); *p* values derived using the Wilcoxon rank-sum test

For other characteristics, p values were derived using chi-squared tests

Muscle weakness was defined using low grip strength (< 26 kg for men, < 16 kg for women)

^+^*p* value derived using Fisher’s exact test

### Assessing Healthy Participant Effects in Analysis Sample

Compared to the 2555 participants who attended the HCS baseline clinic but were not included in the analysis sample, both men and women in the analysis sample had higher baseline self-reported physical activity. Men in the analysis sample were more likely to have never smoked at baseline compared to men who were not included (*p* = 0.04). However, the proportion who were of manual social class (classes IIIM, IV and V) did not differ significantly (*p* > 0.05) between the two groups; this was the case among men and women. Descriptive statistics for these HCS baseline characteristics, according to muscle strength in 2011, are presented in Table 1.

### Health and Social Care Costs for Participants With and Without Muscle Weakness

**Fig. 1:**
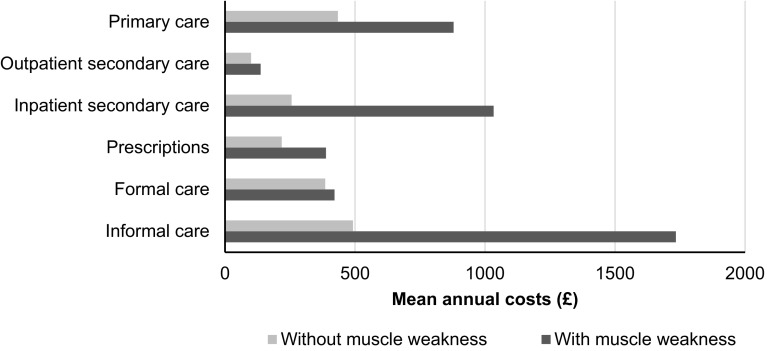
Annual costs per person for different uses of health and social care according to muscle strength. Muscle weakness was defined using low grip strength (< 26 kg for men, < 16 kg for women)

Estimated annual costs per person for different uses of health and social care according to muscle strength are presented in Table 2 and Fig. 1. For each type of health and social care use, costs were greater for individuals with muscle weakness compared to those without. Mean yearly total costs for participants with muscle weakness was £4592 (95% confidence interval: £2962–£6221), with informal care, inpatient secondary care and primary care being responsible for 38%, 23% and 19% of their total costs, respectively. For participants without muscle weakness, total costs were £1885 (£1542–£2228) and their three highest cost categories were informal care (26%), primary care (23%) and formal care (20%). Details of estimated costs by specific classification such as health care specialist consulted, procedure classification or prescription group within each cost category are summarised in Online Appendix 3.

**Table 2:** Estimated annual cost per person by cost component according to muscle strength

Cost component	Without muscle weakness ^a^			With muscle weakness ^a^			*p*
	Mean	Std. err.	95% confidence interval ^b^	Mean	Std. err.	95% confidence interval ^b^	
Primary care	£434	£31	£372–£495	£879	£204	£469–£1288	< 0.001
Outpatient secondary care	£100	£10	£80–£120	£137	£43	£50–£223	< 0.001
Inpatient secondary care	£256	£56	£145–£367	£1033	£375	£278–£1789	< 0.001
Formal care	£385	£90	£208–£562	£421	£142	£135–£708	< 0.001
Informal care	£492	£95	£306–£679	£1734	£388	£953–£2515	< 0.001
Prescriptions	£218	£29	£161–£276	£388	£187	£10–£766	< 0.001
Total	£1885	£175	£1542–£2228	£4592	£810	£2962–£6221	< 0.001

*p* values were obtained from corresponding univariate generalised linear models using a Poisson family distribution and identity link function with each cost component as the outcome variable and muscle weakness classification as the explanatory variable

^a^Muscle weakness was defined using low grip strength (< 26 kg for men, < 16 kg for women)

^b^Based on observed data and 40 imputed datasets via multiple imputation by chained equations

**Fig. 2:**
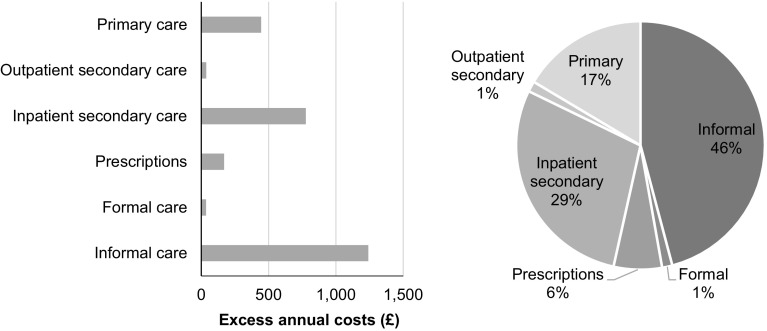
Excess annual costs per person for individuals with muscle weakness compared to those without and proportion of costs according to types of health and social care. Muscle weakness was defined using low grip strength (< 26 kg for men, < 16 kg for women)

The excess annual costs per person for individuals with muscle weakness compared to those without and the proportion of these excess costs, according to types of health and social care use, are presented in Fig. 2. The total excess cost observed for individuals with muscle weakness was £2707 per person per year, with informal care accounting for 46% of total excess costs. After controlling for potential confounders included in Table 1 (age, ever smoking, limiting long-term illness, social class, frailty and disability scores), highly statistically significant differences (*p* < 0.001) in total cost were still observed between individuals with and without muscles weakness based on a GL model using a Poisson family distribution and identity link function.

### Estimate of the Economic Burden Associated with Muscle Weakness in the UK

A calculation of the excess economic burden associated with muscle weakness in the UK is illustrated in Table 3. In mid-2016, the UK population aged 70 years and older was estimated at 8.2 million [23]. Under the assumption that the prevalence of muscle weakness among this group is the same as in HCS (11.1%), a per person per year excess cost of £2707 for muscle weakness results in an annual excess cost associated with muscle weakness of approximately £2.5 billion for the use of health and social care; corresponding costs for health care alone (excluding formal and informal care) were around £1.3 billion.

**Table 3:** Calculation of the excess economic burden associated with muscle weakness in the UK

UK population	65,648,100
UK population aged 70 years and older	8,177,500
UK population aged 70 years and older with muscle weakness*	8,177,500 × 11.1% = 907,703
Excess economic burden for health care in the UK	907,703 × £1429 = £1.30 billion
Excess economic burden for health and social care in the UK	907,703 × £2707 = £2.46 billion

*11.1% of the Hertfordshire Cohort Study participants had muscle weakness using the FNIH criteria (< 26 kg for men and < 16 kg for women). It is assumed that this prevalence is similar in the UK

Population estimates according to the Office for National Statistics

Muscle weakness was defined using low grip strength (< 26 kg for men, < 16 kg for women)

### Sensitivity Analyses

Using grip strength cut-points proposed by Lauretani (< 30 kg for men, < 20 kg for women), 118 (26.7%) participants had low grip strength and the annual per person excess costs were £1256; the corresponding figures for the lowest sex-specific decile approach were 46 (10.4%) participants and £2670. Highly statistically significant differences in total costs were obtained regardless of the grip strength criterion used (*p* < 0.001).

## Discussion

Among HCS participants, the excess economic burden associated with muscle weakness, using FNIH thresholds (< 26 kg for men, < 16 kg for women), was estimated at £2707 per person per year; this results in an estimated total annual excess cost in the UK of £2.5 billion. Informal care was the largest contributor to these excess costs, followed by inpatient secondary care.

These findings have several important implications. They demonstrate that costs associated with muscle weakness represent an important proportion of the health and social care budgets in the UK which are projected to increase in the future due to the ageing population. Furthermore, these results demonstrate that a large proportion of these costs fall on family and friends in the form of informal care, resulting in even higher costs for the state if family and friends were not able to assist with care.

Our results are similar to the findings of a cost-of-illness study in the Czech Republic [8] which reported higher annual direct and indirect health care costs of €564 per person for individuals with weak grip strength (< 26 kg among men, < 16 kg among women) compared to those without. A study among community-dwelling older people in the Netherlands reported significantly higher costs among participants with EWGSOP sarcopenia (€4325 per person per three months, 95% CI €3198–€5471) compared to those without (€1533, 95% CI €1153–€1912) with residential care being a main driver of costs; however, differences in costs between sarcopenics and age- and sex-matched non-sarcopenics were not significant [24]. Annual direct medical costs attributable to sarcopenia were estimated in the United States at around $18.5 billion in 2000, reflecting annual per person excess costs associated with sarcopenia of $860 among men and $933 among women [7]. This was calculated by estimating the health care cost of disability from national surveys and then estimating the proportion of this cost which was due to sarcopenia by examining the extent to which sarcopenia increases the risk of physical disability. Although the costs per person differ between these studies, probably due to the different methods and unit costs used and depending on whether the condition was muscle weakness or sarcopenia, the wider literature supports the substantial economic burden associated with muscle weakness and sarcopenia in Western populations.

Previous literature has also demonstrated the substantial impact of sarcopenia and muscle weakness on direct hospitalisation costs. Among a study of hospitalised patients in Portugal, EWGSOP sarcopenia increased hospitalisation costs by €1240 (95% CI €596–€1887) for patients < 65 years and €721 (95% CI €13–€1429) for patients aged ≥ 65 years [25]. In another study of hospitalised patients in Portugal, increased risks of high hospitalisation costs were observed for people with EWGSOP sarcopenia (OR = 5.70, 95% CI 1.57–20.71) and low grip strength (OR = 2.40, 95% CI 1.12–5.15) compared to those without these conditions [26]. Among a study of patients who underwent radical gastrectomy for gastric cancer in China, the hospital costs, duration of stay and number of complications increased with increasing severity of EWGSOP sarcopenia (pre-sarcopenia, sarcopenia and severe sarcopenia) [27].

This study has some limitations. Firstly, a healthy responder bias has been observed in HCS and attrition across the various waves of follow-up could have resulted in additional selection effects. However, baseline participants remained broadly comparable with participants in the nationally representative Health Survey for England [9] and examining participant characteristics according to inclusion status across the study revealed no major differences. Secondly, health care costs were estimated based on the conservative assumption that, when the frequency of visits and consultations reported by study participants within the last month was once a week or higher, the frequency would have built up to that level and not have been constant at the reported frequency during the whole year. Regarding prescription costs, net ingredient costs by quantity were used as it is the main component of the cost of drugs to the NHS. However, our analysis does not account for the discount percentage received by pharmacists or the container allowance which both influence the total cost to the NHS. Despite this, these two costs may nearly offset each other, meaning ours should be a reliable estimate of prescription costs. Thirdly, direct assessment of muscle mass was not available for the analysis sample, preventing a derivation of sarcopenia status; instead we characterised muscle weakness using the FNIH criteria on grip strength. A final limitation is that this study is observational and hence estimates were based on the costs estimated for study participants with and without muscle weakness. Therefore, interventions to improve muscle strength among participants with muscle weakness, to the point where they do not have muscle weakness according to the definition used in our study, may not significantly reduce costs as these participants are likely to have poorer health compared to those without muscle weakness. Nevertheless, there is evidence of gains in function and independence in intervention studies that have promoted muscle strength [28], which are likely to have little effect on health care costs but potentially sizeable implications by reducing dependence of people with sarcopenia or muscle weakness on informal care, hence lowering broader societal costs. Further research is therefore required on whether lifestyle interventions reduce sarcopenia or muscle weakness among older people and result in reductions in health and social care use.

## Conclusion

This is the first study to show that muscle weakness in older people is associated with significant excess annual costs for health and social care in the UK of around £2.5 billion. These costs are projected to increase in the future due to the ageing population. Lifecourse interventions to reduce the prevalence of muscle weakness among older people are likely to have a substantial beneficial impact on the cost of health and social care in the UK.

## Electronic supplementary material

Below is the link to the electronic supplementary material.

Supplementary material 1 (DOCX 27 KB)
