# Supplementary material for: Health Care Costs Associated With Muscle Weakness: A UK Population-Based Estimate
Source: Calcif Tissue Int. 2018 Sep 22;104(2):137–44. doi: 10.1007/s00223-018-0478-1 (PMC6330088; doi:10.1007/s00223-018-0478-1)
Supplement: Supplementary file 1 — Supplementary material 1 (DOCX 27 KB) [file 223_2018_478_MOESM1_ESM.docx]

**Article title:** Health care costs associated with muscle weakness: a UK population-based estimate

**Journal:** Calcified Tissue International and Musculoskeletal Research

**Authors**: R Pinedo-Villanueva, LD Westbury, HE Syddall, MT Sanchez-Santos, EM Dennison, SM Robinson, C Cooper (corresponding author)

**Affiliations and e-mail address for corresponding author**:

MRC Lifecourse Epidemiology Unit, University of Southampton, Southampton, UK

NIHR Southampton Biomedical Research Centre, University of Southampton and University Hospital Southampton NHS Foundation Trust, Southampton, UK

NIHR Musculoskeletal Biomedical Research Centre, University of Oxford, Oxford, UK

cc@mrc.soton.ac.uk

**Appendix 1**

*Ascertainment of health and social care use*

Questionnaire items asking participants about the number of visits made, and home visits by a general practitioner (GP) and nurse during the previous month were used to estimate resource use for primary care consultations. Yearly estimates were produced based on response options for the previous month as follows: “never” = 0; “once per month” = 12; “twice per month” = 24; “once a week” = 30 (assuming four monthly for first three months, and twice a month thereafter); “three times per week” = 54 (assuming 12 for the first three months, and twice a month thereafter); and “daily” = 72 (assuming 30 for first month, three times weekly for the following two months, and twice a month thereafter).

Use of outpatient secondary care consultations was obtained from an item in the questionnaire asking participants to report on the number of times they had seen, either at the hospital or at home and during the previous year, a rheumatologist, orthopaedic surgeon, A&E doctor/traumatologist, physiotherapist, and a podiatrist. Hospitalisations were reported by participants in an open-question item where dates were reported in some but not all cases. As it was not possible to ascertain whether all reported hospitalisations occurred during the prior year, we conducted the base case analysis including those operations with dates falling within the previous year, and a sensitivity analysis excluding those which, based on their reported dates, were performed more than one year prior. Sensitivity analysis results showed proportionally similar higher costs for both groups with no significant difference in the hospitalisations excess cost associated with muscle weakness (£39 or 5% lower in the sensitivity analysis). Baseline values were used for the full analysis.

Medications were reported by participants including dosage, frequency (daily, weekly, or “as needed”, which was assumed in a first instance to be every two weeks), and how long each medication had been taken. Three different response levels were available for duration, assumed to correspond to the following number of days within the previous year: “less than one month” = 15 days; “one month until one year” = 182 days; “over one year” = 365 days. When participants reported taking the medication “as needed”, a conservative assumption was made that the medication would be taken once a fortnight. Based on the reported frequency and duration that the medication was taken, a total yearly quantity was produced for each product reported by each participant in the corresponding unit (i.e. tablet, capsule, ml, gr, etc.). Where the unit reported did not match the unit in which unit prices were available, conversions were made to match the former to the latter (e.g. 5 ml in 1 teaspoon, 15 ml in 1 tablespoon, 20 ml in 1 drop). When annual estimated cost per medication exceeded £5,000, the case was reviewed by a consultant physician to confirm whether general assumptions were appropriate, and if not, to apply new ones according to the specific drug and patient profile.

Information on both formal and informal care was collected from questionnaire items where participants were asked whether they received formal or informal care at home in the last year, and how often care was received. When asked about formal care, the questionnaire also requested an indication of whether it was provided by Social Services or privately. Response options were once a month; twice a month; once per week; three times per week (assumed to be equivalent to 104 days of help, obtained from the first six months of help at three times per week, and the following six months at once per week); and daily (assumed to be equivalent to 252 days of help, from six months of daily help, and six months at three times per week). In the case of informal help, respondents were also asked who provided the help: wife or husband, son/daughter, other relatives, neighbours, or friends.

**Appendix 2**

|  | **NHS cost** | **Note** |
| --- | --- | --- |
| **GP visit** | £65 | Per patient contact lasting 17.2 minutes |
| **GP home** | £89 | Per patient contact lasting 11.7 minutes plus an average of 12 minutes’ travel time |
| **Nurse visit** | £27 | Cost per hour (£43) x 37.5 working hours per week divided by average of 60 patient consultations per week |
| **Nurse home** | £38 | Mean cost for a face-to-face contact in district nursing services |

Source: Curtis, L. and A. Burns (2015). Unit Costs of Health and Social Care 2015, Personal Social Services Research Unit, University of Kent, Canterbury.

|  | **NHS cost** | **Note** |
| --- | --- | --- |
| **Rheumatologist** | £143 | Hospital Associate specialist: per hour |
| **Orthopaedic surgeon** | £113 | Hospital Associate specialist: per hour |
| **A&E doctor** | £141 | Hospital Associate specialist: per hour |
| **Physiotherapist hospital** | £34 | Mean cost for a non-consultant-led (non-admitted) follow-up physiotherapy attendance |
| **Physiotherapist home** | £41 | As hospital attendance plus an average of 12 minutes travel time per visit |
| **Podiatrist hospital** | £34 | Same as physiotherapist |
| **Podiatrist home** | £41 | Same as physiotherapist |

Sources: Reference Cost Collection: National Schedule of Reference Costs - Year 2014-15; and Curtis, L. and A. Burns (2015). Unit Costs of Health and Social Care 2015, Personal Social Services Research Unit, University of Kent, Canterbury.

|  | **Cost** | **Note** |
| --- | --- | --- |
| **Social services** | £45 | Local authority day care for older people: £45 per client session lasting 3.5 hours |
| **Private** | £69 | Social care support for older people: Home care: Average weekly costs: £178 (9 hours per week). Prorated to 3.5 hours |

Source: Curtis, L. and A. Burns (2015). Unit Costs of Health and Social Care 2015, Personal Social Services Research Unit, University of Kent, Canterbury.

|  | **Cost** | **Note** |
| --- | --- | --- |
| **Help provided by son or daughter** | £42 | Average weekly earnings during 2015 was £483. Assuming full time of 40 hours and 3.5 hours informal help per day session (same as formal care) |
| **Help provided by any other than son or daughter** | £23 | For 2015, National Minimum Wage was £6.70 per hour. Assuming 3.5 hours’ informal help per day session (same as formal care) |

Sources: Office of National Statistics. Time series: LMSB SA AWE total pay WE. Available from: https://www.ons.gov.uk/employmentandlabourmarket/peopleinwork/earningsandworkinghours/timeseries/kab9/emp; and Gov UK. National Minimum Wage and National Living Wage rates. Available from: https://www.gov.uk/national-minimum-wage-rates.

**Appendix 3**

**Primary care consultations**

|  | **Estimated number of yearly consultations per person** | | **Estimated  annual cost per person** | |
| --- | --- | --- | --- | --- |
|  | **Mean** | **SD** | **Mean** | **SD** |
| **Without muscle weakness** |  |  |  |  |
| GP |  |  | £329.8 | £538.6 |
| *GP practice visit* | 5.0 | 8.2 |  |  |
| *GP home visit* | 0.1 | 0.9 |  |  |
| Nurse |  |  | £103.9 | £228.4 |
| *GP practice visit* | 3.6 | 7.6 |  |  |
| *Nurse home visit* | 0.2 | 2.8 |  |  |
| **Total** |  |  | **£433.7** |  |
| **Total post-imputation*** | |  | **£433.5** | **£615.5** |
|  |  |  |  |  |
| **With muscle weakness** |  |  |  |  |
| GP |  |  | £592.6 | £837.7 |
| *GP practice visit* | 7.1 | 10.3 |  |  |
| *GP home visit* | 1.5 | 5.3 |  |  |
| Nurse |  |  | £286.0 | £742.5 |
| *GP practice visit* | 4.4 | 9.9 |  |  |
| *Nurse home visit* | 1.1 | 5.4 |  |  |
| **Total** |  |  | **£878.6** | **£1,424.6** |
| * One individual reported missing number of consultations and their costs were  imputed using a generalised linear model | | | | |
|  | | | | |
| Muscle weakness was defined using low grip strength (<26kg for men, <16kg for women) | | | | |

**Secondary care consultations**

|  | **Number of yearly consultations per person** | | **Annual cost per person** | |
| --- | --- | --- | --- | --- |
|  | **Mean** | **SD** | **Mean** | **SD** |
| **Without muscle weakness** |  |  |  |  |
| Rheumatologists | 0.04 | 0.4 | £6.3 | £57.3 |
| Orthopaedic surgeons | 0.21 | 0.75 | £23.5 | £84.6 |
| Trauma doctors | 0.15 | 0.43 | £21.1 | £60.4 |
| Physiotherapist |  |  | £17.0 | £77.3 |
| *Hospital visit* | 0.47 | 2.2 |  |  |
| *Home visit* | 0.03 | 0.4 |  |  |
| Podiatrist |  |  | £31.5 | £90.8 |
| *Hospital visit* | 0.29 | 1.4 |  |  |
| *Home visit* | 0.53 | 2.0 |  |  |
| **Total** |  |  | **£99.4** |  |
| **Total post-imputation*** | |  | **£99.7** | **£201.4** |
|  |  |  |  |  |
| **With muscle weakness** |  |  |  |  |
| Rheumatologists | 0.14 | 0.5 | £20.4 | £71.4 |
| Orthopaedic surgeons | 0.18 | 0.7 | £20.7 | £78.5 |
| Trauma doctors | 0.14 | 0.4 | £20.1 | £57.4 |
| Physiotherapist |  |  | £17.5 | £70.2 |
| *Hospital visit* | 0.49 | 2.1 |  |  |
| *Home visit* | 0.02 | 0.1 |  |  |
| Podiatrist |  |  | £57.9 | £258.1 |
| *Hospital visit* | 1.41 | 7.4 |  |  |
| *Home visit* | 0.24 | 1.4 |  |  |
| **Total** |  |  | **£136.6** | **£299.5** |
| * Nine people reported missing number of consultations and their costs were imputed using the arithmetic mean of non-missing responses for the missing components after regression imputation models failed to converge | | | | |
|  | | | | |
| Muscle weakness was defined using low grip strength (<26kg for men, <16kg for women) | | | | |

**Prescriptions**

| **British National Formulary classification** | **Annual cost per person without muscle weakness** | | **Annual cost per person with muscle weakness** | |
| --- | --- | --- | --- | --- |
|  | **Mean** | **Std. Err.** | **Mean** | **Std. Err.** |
| Gastro-Intestinal | £5.0 | £5.9 | £11.7 | £6.4 |
| Cardiovascular | £47.0 | £5.2 | £51.7 | £15.6 |
| Respiratory | £29.1 | £7.3 | £30.0 | £20.8 |
| Central Nervous System | £16.7 | £4.5 | £21.6 | £9.6 |
| Infections | £0.8 | £0.3 | £11.7 | £30.2 |
| Endocrine System | £49.8 | £21.1 | £30.5 | £82.1 |
| OB/GY/UT disorders | £6.0 | £2.7 | £1.8 | £1.4 |
| Malignant disease | £5.6 | £3.5 | £161.2 | £161.2 |
| Nutrition & blood | £26.1 | £7.9 | £14.3 | £4.7 |
| Musculoskeletal | £6.3 | £1.8 | £40.5 | £28.2 |
| Eye | £9.4 | £5.6 | £0.4 | £0.6 |
| Ear, Nose and Throat | £2.6 | £1.3 | £1.7 | £1.4 |
| Skin | £1.9 | £1.3 | £2.0 | £1.8 |
| Other | £0.3 | £0.3 | - | - |
| Appliances | £0.6 | £0.3 | £6.0 | £6.0 |
| Incontinence* | £10.9 |  | £2.7 | £2.7 |
| **Total** | **£218.1** |  | **£387.8** |  |
| Note: Estimates produced after applying multiple imputation by chained equations methods. In groups where no prescriptions were reported, no values are shown and £0 was added to their overall annual costs.  * Standard errors could not be calculated due to inconsistency amongst multiple imputation datasets for the prescription group amongst study participants without muscle weakness. The mean value was estimated by subtracting all other groups means from the total cost for prescriptions. | | | | |
|  | | | | |
| Muscle weakness was defined using low grip strength (<26kg for men, <16kg for women) | | | | |

**Inpatient secondary care**

| **Procedure classification** | **Annual cost per person without muscle weakness** | | **Annual cost per person with muscle weakness** | |
| --- | --- | --- | --- | --- |
|  | **Mean** | **SD** | **Mean** | **SD** |
| Endocrine | - | - | £44.0 | £307.9 |
| Rheumatological | £7.7 | £152.5 | - | - |
| Orthopaedic | £96.3 | £662.2 | £131.2 | £707.2 |
| Genitourinary | £16.5 | £133.3 | £61.8 | £265.3 |
| Haematological | £1.3 | £25.0 | - | - |
| Neurological | - | - | - | - |
| Cardiovascular | £29.3 | £460.5 | £260.3 | £1,304.6 |
| Gastrointestinal | £9.8 | £137.5 | £281.5 | £1,213.7 |
| Ophthalmology | £14.1 | £98.4 | £14.5 | £101.6 |
| ENT | £18.6 | £230.5 | - | - |
| Respiratory | £7.6 | £151.0 | - | - |
| Dermatology | £14.9 | £185.3 | £120.8 | £599.6 |
| General Surgical | £39.7 | £467.0 | £119.3 | £834.9 |
| Other | £0.5 | £10.3 | - | - |
| **Total** | **£256.3** |  | **£1,033.4** |  |
| Note: In operation classification groups where no procedures were reported, no values are shown and £0 was added to their overall annual costs. | | | | |
|  | | | | |
| Muscle weakness was defined using low grip strength (<26kg for men, <16kg for women) | | | | |

**Formal care**

|  | **Without  muscle weakness** | | **With  muscle weakness** | |
| --- | --- | --- | --- | --- |
|  | **n** | **(%)** | **n** | **(%)** |
| **Received formal care at home?** | |  |  |  |
| No | 349 | (88.8) | 40 | (81.6) |
| Yes | 44 | (11.2) | 9 | (18.4) |
| **If so, who provided it?** | |  |  |  |
| Social services | 4 | (9.1) | 1 | (11.1) |
| Private | 40 | (90.9) | 8 | (88.9) |
| **If so, how frequent?** | |  |  |  |
| Daily | 4 | (9.1) | 0 | (0) |
| Once week | 19 | (43.2) | 4 | (44.4) |
| Twice month | 7 | (15.9) | 4 | (44.4) |
| Once month | 8 | (18.2) | 1 | (11.1) |
| *Missing* | *6* | *(13.6)* | *0* | *(0)* |
|  |  |  |  |  |
| Muscle weakness was defined using low grip strength (<26kg for men, <16kg for women) | | | | |

**Informal care**

|  | **Without  muscle weakness** | | **With  muscle weakness** | |
| --- | --- | --- | --- | --- |
|  | **n** | **(%)** | **n** | **(%)** |
| **Received informal help at home?** | |  |  |  |
| No | 356 | (91.0) | 31 | (63.3) |
| Yes | 35 | (9.0) | 18 | (36.7) |
| *Missing* | *2* | *(0.5)* | *0* | *(0)* |
| **If so, who provided it?** | |  |  |  |
| Spouse | 11 | (31.4) | 13 | (72.2) |
| Child | 22 | (62.9) | 4 | (22.2) |
| Other relative | 1 | (2.9) | 0 | (0) |
| Friend | 1 | (2.9) | 0 | (0) |
| Neighbour | 0 | (0) | 1 | (5.6) |
|  |  |  |  |  |
| Muscle weakness was defined using low grip strength (<26kg for men, <16kg for women) | | | | |
